# Heteroepitaxial Growth of T-Nb_2_O_5_ on SrTiO_3_

**DOI:** 10.3390/nano8110895

**Published:** 2018-11-01

**Authors:** Jos E. Boschker, Toni Markurt, Martin Albrecht, Jutta Schwarzkopf

**Affiliations:** Leibniz-Institut für Kristallzüchtung, Max-Born-Straße 2, D-12489 Berlin, Germany; toni.markurt@ikz-berlin.de (T.M.); martin.albrecht@ikz-berlin.de (M.A.); jutta.schwarzkopf@ikz-berlin.de (J.S.)

**Keywords:** niobium oxide, epitaxy, metal-organic chemical vapor deposition

## Abstract

There is a growing interest in exploiting the functional properties of niobium oxides in general and of the T-Nb_2_O_5_ polymorph in particular. Fundamental investigations of the properties of niobium oxides are, however, hindered by the availability of materials with sufficient structural perfection. It is expected that high-quality T-Nb_2_O_5_ can be made using heteroepitaxial growth. Here, we investigated the epitaxial growth of T-Nb_2_O_5_ on a prototype perovskite oxide, SrTiO_3_. Even though there exists a reasonable lattice mismatch in one crystallographic direction, these materials have a significant difference in crystal structure: SrTiO_3_ is cubic, whereas T-Nb_2_O_5_ is orthorhombic. It is found that this difference in symmetry results in the formation of domains that have the T-Nb_2_O_5_ c-axis aligned with the SrTiO_3_ <001>_s_ in-plane directions. Hence, the number of domain orientations is four and two for the growth on (100)_s_- and (110)_s_-oriented substrates, respectively. Interestingly, the out-of-plane growth direction remains the same for both substrate orientations, suggesting a weak interfacial coupling between the two materials. Despite challenges associated with the heteroepitaxial growth of T-Nb_2_O_5_, the T-Nb_2_O_5_ films presented in this paper are a significant improvement in terms of structural quality compared to their polycrystalline counterparts.

## 1. Introduction

Niobium oxides have been studied for a long time [1,2] and are of technological interest for different potential applications. For example, niobium oxides are very attractive for energy storage applications due to their intercalation pseudocapacitance [3,4]. In addition, niobium oxides can be used as selectors or data storage elements in future nonvolatile memory applications [5,6]. The most thermodynamically stable niobium oxide is niobium pentoxide (Nb_2_O_5_) [1,2]. Nb_2_O_5_ has many different polymorphs that are discussed elsewhere in detail [1,2]. The most important polymorphs are the low-temperature polymorph, T-Nb_2_O_5_, which has an orthorhombic symmetry with a superstructure [7], and the high-temperature polymorph, H-Nb_2_O_5_, which is the most stable polymorph and has a monoclinic symmetry [8]. Of these two, T-Nb_2_O_5_ is the most relevant for practical applications, because it can be formed at lower temperatures and exhibits intercalation pseudocapacitance [3,4]. Unfortunately, the availability of niobium oxides with high structural perfection, such as bulk single crystals or as a single-crystal layer, is limited, to date. This makes it difficult to determine the fundamental functional properties of niobium oxides, such as anisotropic optical constants, energetic levels of relevant point defects, or anisotropies in electrical conduction. The determination of these properties is highly desirable in order to improve the functionality of niobium oxides for applications.

Heteroepitaxial film growth is interesting in this respect, because it enables the fabrication of high-quality material on foreign substrates and the determination of its properties. Even though polycrystalline T-Nb_2_O_5_ films are well studied (see, for example, References [9,10]), epitaxial T-Nb_2_O_5_ thin films are not widely available nor studied. Hence, for the full exploitation of their functional properties, the growth of epitaxial T-Nb_2_O_5_ is urgently needed.

**Figure 1:**
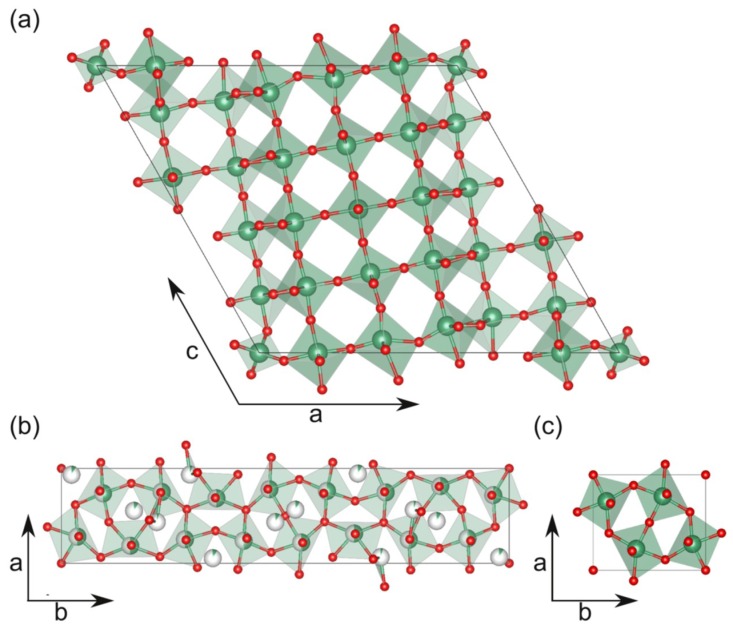
Structural models of (**a**) H-Nb_2_O_5_ and (**b**,**c**) T-Nb_2_O_5_. The model in (**b**) is adapted from Kato et al. [11], and the model in (**c**) is adapted from Lee et al. [12].

Both the T-Nb_2_O_5_ and the H-Nb_2_O_5_ polymorphs consist of oxygen octahedra that are connected by their corners, as well as by their edges, as seen in Figure 1b,c and Figure 1a, respectively. For T-Nb_2_O_5_, the structure determined by Kato et al. [11] is shown in Figure 1b, whereas a simplified model of T-Nb_2_O_5_ based on Lee et al. [12] is shown in Figure 1c. These configurations result in nanometer-sized unit cells with orthorhombic and monoclinic symmetry, respectively. Due to the unique symmetries of these polymorphs, no symmetry-matched substrate is available for heteroepitaxy. Advantageously, the lattice parameter of SrTiO_3_ (a = 3.905 Å) is well matched with the c-lattice parameter of T-Nb_2_O_5_ (3.938 Å) and b-lattice parameter of H-Nb_2_O_5_ (3.824 Å), resulting in lattice mismatches of −0.8% and +2.1%, respectively. Presently, it is unclear how the presence of this symmetry mismatch in combination with a good lattice match affects the growth and the structural properties of T-Nb_2_O_5_ thin films on SrTiO_3_. In order to investigate this, we have grown epitaxial Nb_2_O_5_ thin films on SrTiO_3_ using metal-organic chemical vapor deposition (MOCVD). First, the deposition regimes for the growth of T-Nb_2_O_5_ and H-Nb_2_O_5_ were identified. Next, we focused on the influence of the symmetry mismatch between T-Nb_2_O_5_ and SrTiO_3_ on the domain formation, because the T-Nb_2_O_5_ phase is the technologically most relevant phase. This influence was studied by employing (100)_s_- and (110)_s_-oriented SrTiO_3_ substrates. Finally, the microstructure of the T-Nb_2_O_5_ thin films was analyzed using transmission electron microscopy (TEM).

## 2. Materials and Methods

Thin films were grown by means of liquid-delivery spin metal-organic chemical vapor deposition (MOCVD). For the deposition, a 0.01 M or 0.005 M solution of niobium ethoxide (Nb(EtO)_5_) in toluene was used. The solution was evaporated in an evaporator with a temperature of 180 °C at a rate of approximately 0.45 mL/min. Using Ar as a carrier gas, the evaporated solution was fed into the MOCVD reactor. A rotation speed of the carrier of 750 rounds per minute was employed. Using a 0.01 M solution, a growth rate of approximately 1.5 nm/min was achieved. The (100)_s_ SrTiO_3_ substrates were etched with buffered HF and subsequently annealed for 1 h at 1100 °C in a pure oxygen flow prior to the deposition. The (110)_s_ SrTiO_3_ substrates were annealed for 1 h at 1000 °C in pure oxygen. The surface morphology of the substrates and films was studied using a Bruker Icon atomic force microscope (AFM, Billerica, MA, USA) operated in PeakForce Tapping^®^ mode. The structural properties were studied by high-resolution X-ray diffraction (HRXRD) with a Bruker D8 Discover system (Billerica, MA, USA) using Cu Kα_1_ radiation. In order to differentiate between film and substrate, the indices film (“f”) and substrate (“s”) are added to the Miller indices of the film and substrate, respectively. The film thickness was determined by spectral ellipsometry using a Horiba Jobin Yvon MM-16 (Kyoto, Japan).

Cross-sectional transmission electron microscopy (TEM) investigations were performed with an aberration-corrected FEI Titan 80–300 (Hilsboro, OR, USA) operating at 300 kV. For high-resolution TEM (HRTEM) imaging, a spherical aberration of C_s_ = −10 µm was used, and all other aberrations were corrected to a minimum. TEM samples were prepared by mechanical polishing using diamond lapping foils down to a thickness of approximately 10 µm. Final thinning to electron transparency was done by argon ion milling, with accelerating voltages decreasing stepwise from 4 kV to 0.2 kV using a GATAN precision ion polishing system (PIPS, Pleasanton, CA, USA). The structural models presented in Figure 1and Figure 6 were made using VESTA [13].

## 3. Results and Discussion

### 3.1. Determination of the Growth Window

**Figure 2:**
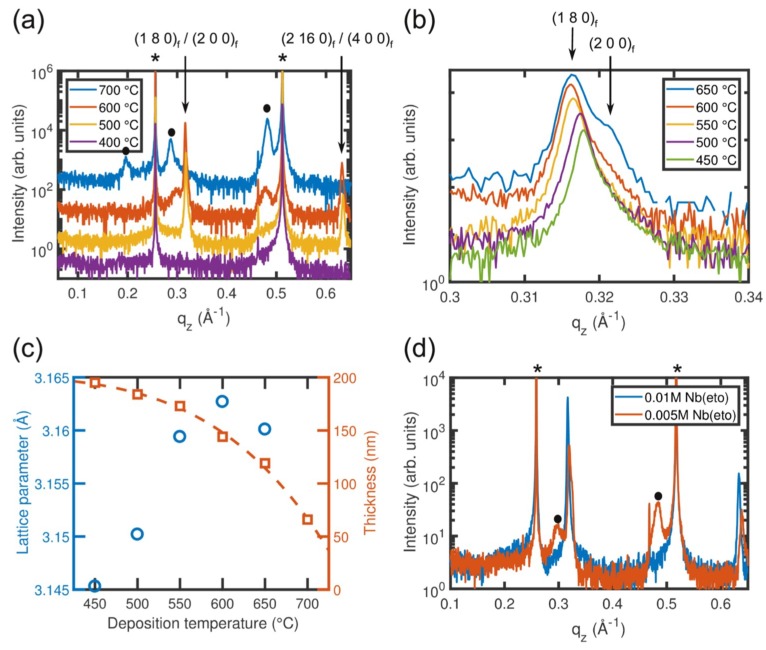
Wide range X-ray diffraction patterns of Nb_2_O_5_ thin films on SrTiO_3_ (100)_s_ grown at different temperatures (**a**) and using different niobium ethoxide concentrations, i.e., different film growth rates, at a fixed growth temperature of 600 °C (**d**). (**b**) θ/2θ scans with higher magnification for the films grown at 450 °C, 500 °C, 550 °C, 600 °C, and 650 °C. (**c**) (180) Lattice parameter of T-Nb_2_O_5_ and the film thickness as a function of the deposition temperature. The dashed line in (**c**) shows the fitting of the data. The asterisks in (**a**,**d**) indicate the positions of the substrate peaks, whereas the circles refer to the peaks corresponding to the H-Nb_2_O_5_ phase.

Initially, Nb_2_O_5_ films were grown on (100)_s_ SrTiO_3_ substrates employing substrate temperatures between 400 °C and 750 °C with intervals of 50 °C in order to assess the growth window. Figure 2a shows the wide-range HRXRD profiles of films grown at temperatures of 400 °C, 500 °C, 600 °C, and 700 °C that capture the most important observations. All diffraction patterns contain sharp peaks at approximately 0.255 Å^−1^ and 0.51 Å^−1^ that belong to the (100)_s_ and (200)_s_ diffraction peaks of the SrTiO_3_ substrate, respectively (marked with an asterisk). The small peak observed at 0.46 Å^−1^ is a measurement artifact caused by Cu Kß radiation. The other Bragg peaks, which are visible in Figure 2a, are attributed to the niobium oxide films. Significant differences can be seen between the diffraction profiles of films grown at different temperatures.

**Figure 3:**
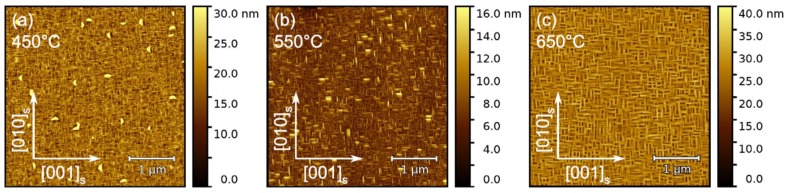
Surface morphology of T-Nb_2_O_5_ films grown on (100)_s_-oriented SrTiO_3_ using growth temperatures of (**a**) 450 °C, (**b**) 550 °C and (**c**) 650 °C, as determined by atomic force microscopy (AFM). The data reveal an increase in the grain size with increasing temperature. The arrows indicate the crystallographic directions of the substrate.

No diffraction peaks are observed for the film grown at 400 °C, indicating an amorphous film structure. With increasing substrate temperature, the occurrence of Bragg peaks indicates the formation of a crystalline phase. However, depending on temperature, differences in the diffraction pattern are visible: (1) The diffraction patterns of the films grown between 450 °C and 650 °C exhibit a strong peak at q_z_ ≈ 0.316 Å^−1^ corresponding to a lattice spacing of about 3.165 Å. This spacing is in reasonable agreement with the (180)_f_ lattice spacing of T-Nb_2_O_5_ (3.15 Å) [PDF 00-027-1313]. A higher magnification in the q_z_ range between 0.3 Å^−1^ and 0.34 Å^−1^ (Figure 2b) shows that, additionally, a minor contribution at q_z_ ≈ 0.322 Å^−1^ occurs that is attributed to the (200)_f_ reflection of T-Nb_2_O_5_. The intensity of the (200)_f_ reflection is approximately 1% of the (180)_f_ diffraction peak for a deposition temperature of 600 °C and decreases with decreasing deposition temperature. The overall shift of the film peak to lower q_z_ values with increasing deposition temperature indicates that the lattice parameter increases, as shown in Figure 2c. This might be simply explained by an increase in the build-up of compressive strain when cooling the sample to room temperature after growth that is induced by the difference in the thermal expansion coefficient between Nb_2_O_5_ (1.3 × 10^−5^ K^−1^ [14]) and the SrTiO_3_ substrate (3.23 × 10^−5^ K^−1^ [15]). AFM investigations (see Figure 3) show that on (100)_s_ SrTiO_3_ substrates, T-Nb_2_O_5_ grows with a fourfold rotational symmetry with needle-like grains aligned along the [010]s and [001]s directions of the substrate (in the following, the indices “f” and “s” denote the film and the substrate directions, respectively). Moreover, it can be seen that the grain size increases with increasing growth temperature. Such a change in grain size can result in the development of a tensile strain in the thin film that originates during the grain boundary formation [16], which would result in a shift of the (180)_f_ diffraction peak to higher q_z_ values with increasing growth temperature. This is observed for temperatures above 600 °C, suggesting that both the difference in thermal expansion and the domain size contribute to the strain state of the T-Nb_2_O_5_ film.

For the film grown at 700 °C, the T-Nb_2_O_5_ (180)_f_ and (200)_f_ peaks in the HRXRD θ/2θ scan are no longer visible. Instead, three Bragg reflections at 0.197 Å^−1^, 0.287 Å^−1^, and 0.481 Å^−1^ (marked by black circles in Figure 2a) are observed. These three peaks belong to the (301)_f_, (402)_f_, and (703)_f_ diffraction peaks of the high-temperature phase of Nb_2_O_5_ (H-Nb_2_O_5_), respectively [pdf 00-037-1468]. In general, it is observed that the amount of H-Nb_2_O_5_ gradually increases with the deposition temperature: for deposition temperatures of 550 °C and below, no significant contribution of the H-Nb_2_O_5_ phase is observed (for clarity, the diffraction profile of the film grown at 550 °C is not shown in Figure 2a). For films grown between 600 °C and 650 °C, the main phase is T-Nb_2_O_5_, but a small fraction of about 1% of the H-Nb_2_O_5_ phase is already present. Finally, the deposition rate decreases with increasing deposition temperature, as shown in Figure 2c. This is likely caused by thermal desorption. By fitting the data, an activation energy of 0.67 ± 0.1 eV is determined.

Besides the temperature, it was also found that the occurrence of T-Nb_2_O_5_ and H-Nb_2_O_5_ depends on the growth rate. The diffraction profiles of two films grown at 600 °C with different growth rates (controlled by the concentration of niobium ethoxide precursor in toluene) reveals that the peaks corresponding to the H-Nb_2_O_5_ phase are absent and pure T-Nb_2_O_5_ is deposited when a higher growth rate is used, whereas the H-Nb_2_O_5_ polymorph is clearly present for a reduced growth rate (Figure 2d). These observations indicate that the transformation from T-Nb_2_O_5_ to H-Nb_2_O_5_ is a thermally activated process. The transformation thus depends on both the deposition rate and the temperature. The presented results demonstrate that pure T-Nb_2_O_5_ and H-Nb_2_O_5_ phases can be obtained by choosing the appropriate deposition temperatures and growth rates.

### 3.2. Domain Formation of T-Nb_2_O_5_ on (100)_s_- and (110)_s_-Oriented SrTiO_3_

**Figure 4:**
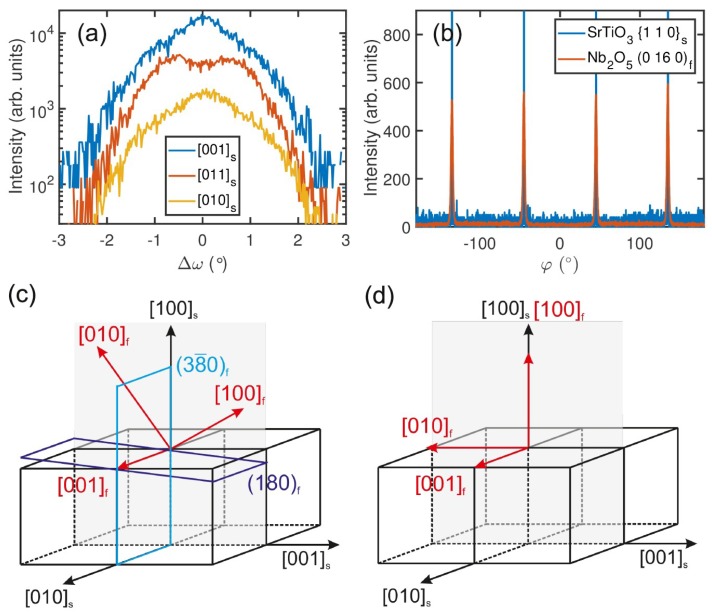
(**a**) ω-scans of the (180)_f_ diffraction peak with the X-ray beam parallel to different in-plane directions of the substrate, as indicated in the inset. (**b**) φ-scans of the SrTiO_3_ {110}_s_ diffraction peaks and the T-Nb_2_O_5_ (0 16 0)_f_ diffraction peak. The observation of four T-Nb_2_O_5_ (0 16 0)_f_ peaks indicates that four grain orientations are present in the film. (**c**,**d**) Schematics of the crystallographic relationship between the SrTiO_3_ substrate and (180)_f_-oriented T-Nb_2_O_5_ and (100)_f_-oriented T-Nb_2_O_5_, respectively. The indices “f” and “s” denote directions corresponding to the film and the substrate, respectively.

In the following, we investigate the effect of lattice parameter mismatch and the symmetry of the substrate on the film growth and domain formation. We first concentrate on the T-Nb_2_O_5_ films on (100)_s_-oriented SrTiO_3_ and analyze the epitaxial relationship in more detail. Figure 4a shows ω-scans of the (180)_f_ diffraction peaks of a T-Nb_2_O_5_ film grown at 600 °C. The shape of the ω-scan depends on the in-plane orientation of the substrate relative to the incident X-ray beam. When the X-ray beam is parallel to the [011]s in-plane direction of the substrate, two peaks are observed at ±0.7°. In the case of X-rays parallel to the [010]s or [001]s in-plane directions of the (100)s SrTiO3 substrate, a maximum at ω = 0° and two shoulders at ω = ±1.0° are observed. The peak splitting points to the formation of domains which are tilted by ±ω = 1.0° with respect to the surface normal toward the [010]s and [001]s directions. In addition, φ-scans were performed in order to study the number of domains in more detail. The φ-scan, presented in Figure 4b, reveals the presence of four (0 16 0)_f_ T-Nb_2_O_5_ peaks aligned with the SrTiO_3_ substrate, indicating that four T-Nb_2_O_5_ domain orientations are present in the film.

In order to understand these observations, the lattice mismatch between the T-Nb_2_O_5_ structure and (100)_s_ SrTiO_3_ surface has to be considered. While the dimensions of the surface unit cell of (100)_s_ SrTiO_3_ is given by 3.905 Å × 3.905 Å, the bulk lattice parameters of the orthorhombic T-Nb_2_O_5_ polymorph amount to *a* = 6.168 Å, *b* = 29.312 Å, and *c* = 3.938 Å [2]. According to the XRD data (Figure 2b), the films contain (200)_f_- and (180)_f_-oriented domains. For both orientations, the *c-*lattice parameter of T-Nb_2_O_5_ can be aligned with one in-plane direction of the (100)_s_ SrTiO_3_ substrate, either along ±[010]s or ±[001]s. A schematic displaying both configurations of the crystallographic arrangement is given in Figure 4c,d. This epitaxial alignment results in a lattice mismatch of only −0.8% in the *c*-direction and is, therefore, expected to be energetically favorable. Since all in-plane variants are equivalent, there are four different in-plane orientations for the domains. This theoretical assumption of the presence of four different in-plane variants with (180)_f_ orientation is in good agreement with the ω- and φ-scans that were performed on the (180)_f_ and (0 16 0)_f_ diffraction peaks, i.e., Figure 4a,b.

The in-plane lattice alignment of the (180)_f_-oriented domains in the direction orthogonal to the *c*-axis cannot be guessed intuitively, because there exists no low-indexed/highly symmetric lattice plane that is both orthogonal to the (001)_f_ and (180)_f_ planes. Instead, it is found that the (3−80)_f_ plane makes an angle of 88.9° with the (180)_f_ plane. Assuming that (3−80)_f_ is parallel with the (001)_s_ plane, the (180)_f_ plane is tilted by α = −1.1° from the [001]s direction of the substrate, as shown schematically in Figure 4c. This tilt angle is in good agreement with the tilt angle determined from the ω-scans in Figure 2a (peak splitting by ∆ω ≈ 2°). Therefore, we conclude that the (3−80)_f_ plane is indeed lying parallel to the (100)_s_ plane of the SrTiO_3_ substrate. Finally, it is noted that the lattice mismatch in the direction orthogonal to the *c*-axis amounts to several percent and leads to a strong lattice relaxation in this direction. 

**Figure 5:**
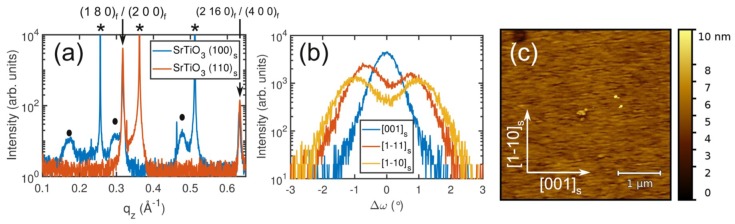
(**a**) Comparison of the XRD profiles of T-Nb_2_O_5_ on (100)_s_ SrTiO_3_ (blue) and (110)_s_ SrTiO_3_ (red). The peaks associated with the substrate and H-Nb_2_O_5_ are marked with an asterisk and filled circles, respectively. (**b**) ω-scans of the (180)_f_ diffraction peak with the X-ray beam parallel to different in-plane directions for T-Nb_2_O_5_ on SrTiO_3_ (110)_s_. (**c**) Surface morphology of T-Nb_2_O_5_ on SrTiO_3_ (110)_s_ film as determined by AFM (**c**). The root mean square surface roughness is 0.7 nm. The crystallographic directions of the substrate are indicated by the arrows.

In contrast to the (100)_s_ SrTiO_3_ surface, the (110)_s_ SrTiO_3_ surface does not exhibit a fourfold rotational symmetry but only a twofold one. The surface unit cell amounts to d_001_ = 3.905 Å and d_1–10_ = 5.522 Å. Similar to the film growth on (100)_s_ SrTiO_3_, only the unit cell dimension of (110)_s_ SrTiO_3_ along the [001]s direction (d_001_ = 3.905 Å) fits to one of the lattice parameters of T-Nb_2_O_5_, namely the *c*-axis (*c* = 3.938 Å). Therefore, the number of domain orientations on SrTiO_3_ (110)_s_ is expected to be reduced with respect to the SrTiO_3_ (100)_s_ surface. Figure 5a shows that a pure T-Nb_2_O_5_ phase was grown on (110)_s_ SrTiO_3_ at 600 °C. A comparison of the films grown on (100)_s_ and (110)_s_ SrTiO_3_ at 600 °C reveals that (i) on SrTiO_3_ (110)_s_, no H-Nb_2_O_5_ phase was additionally observed, and (ii) both films have a preferential (180)_f_ out-of-plane direction, suggesting that this is the preferred growth direction. Furthermore, the (200)_f_ out-of-plane orientation was not observed for films grown on SrTiO_3_(110)_s_ within the experimental resolution. Again, the orientation of the domains in the film was studied by performing XRD ω-scans with the X-ray beam along different in-plane directions of the substrate, as indicated in Figure 5b. The data clearly differ from the case of growth on (100)_s_ SrTiO_3_ substrates, because only a single peak is observed when the X-ray beam is parallel with the [001]s direction. This peak is—however—split for the scans in the [1−11]_s_ and [1−10]_s_ directions, indicating that there are two domain orientations present in films grown on (110)_s_ SrTiO_3_ that have (180)_f_ lattice planes that are tilted by approximately ±1° with respect to the [110]s orientation of the substrate. This tilt angle is the same as that in the case for T-Nb_2_O_5_ grown on (100)_s_ SrTiO_3_. Therefore it is concluded that the (3−80)_f_ plane is parallel with the (1−10)_s_ plane of the SrTiO_3_ substrate. In addition, it was found that the reduction of the number of domain orientations results in a significant change of the surface morphology. Instead of the rough surface with fourfold rotational symmetry that is observed on (100)_s_-oriented substrates, the surface of the film grown on the (110)_s_-oriented SrTiO_3_ reflects the twofold symmetry, as observed by XRD, and has a root mean square surface roughness of 0.7 nm, as can be deduced from the data presented in Figure 5c. 

The presented data thus show that the symmetry mismatch between T-Nb_2_O_5_ and SrTiO_3_ result in the formation of domains with different crystallographic orientations. Interestingly, the in-plane orientation is determined by the alignment of the T-Nb_2_O_5_ c-axis with the <001> directions of the substrate, whereas the out-of-plane direction is the same for thin films grown on (100)_s_- and (110)_s_-oriented SrTiO_3_. The latter could be due to a preferred growth direction and suggest that the structural coupling across the interface is weak, because, in the case of a strong coupling, the film orientation would be determined by the substrate orientation and would thus change when the substrate orientation changes.

### 3.3. TEM Investigations of the Microstructure

**Figure 6:**
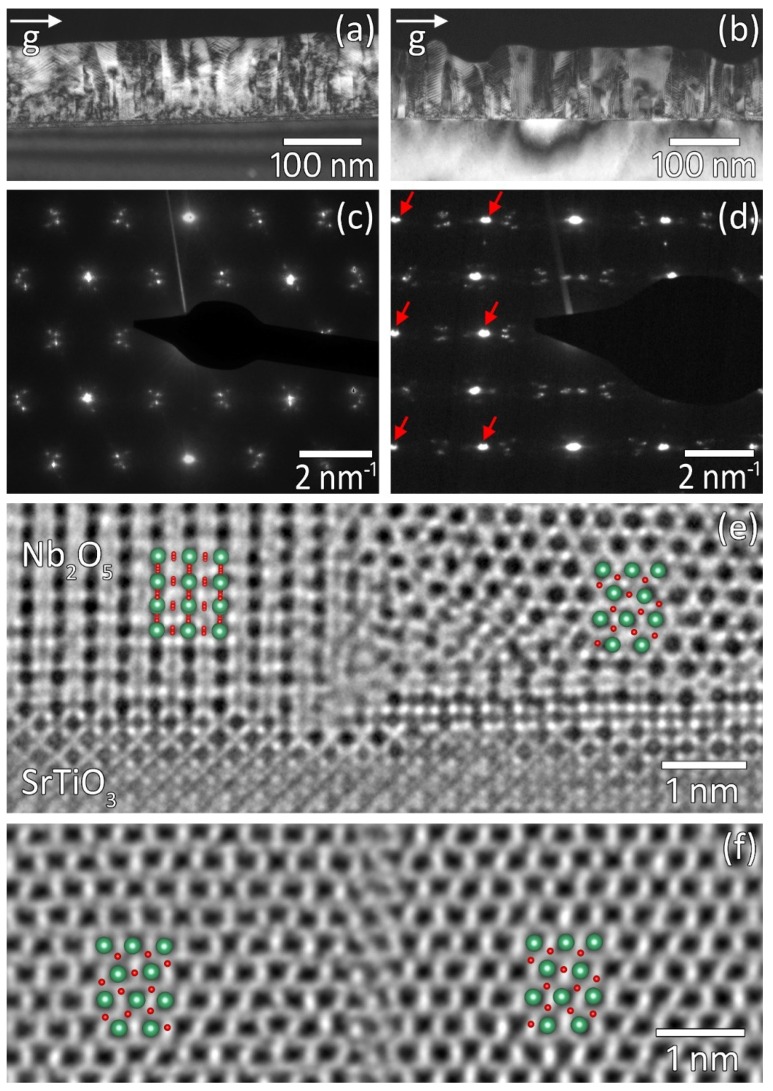
Cross-sectional TEM images of Nb_2_O_5_ films grown on SrTiO_3_. (**a**,**c**,**f**) (110)_s_-oriented substrate, (**b**,**d**,**e**) (100)_s_-oriented substrate. (**a**,**b**) TEM dark field, (**c**,**d**) electron diffraction, and (**e**,**f**) HRTEM image, where the insets show models of T-Nb_2_O_5_ (green and red balls denote Nb and O, respectively).

Up to now, we can summarize that the use of (110)_s_-oriented SrTiO_3_ substrates leads to a reduction in the number of in-plane rotational domain variants in T-Nb_2_O_5_ thin films from four to two. The domains have (180)_f_ out-of-plane orientation with a tilt angle of about ±1° from the [110]s substrate surface normal. In the following, the structural properties of these films are analyzed in greater detail using transmission electron microscopy (TEM). Figure 6 shows cross-sectional TEM images of T-Nb_2_O_5_ films grown on (110)_s_- and (100)_s_-oriented SrTiO_3_ substrates, respectively. In agreement with our XRD measurements, electron diffraction (Figure 6c,d) and HRTEM imaging (Figure 6e,f) reveal that, in both cases, the film grows with (180)_f_ orientation. Abrupt intensity variations within the T-Nb_2_O_5_ film for both substrate orientations in dark-field TEM images recorded close to the [001]s zone axis (see Figure 5a,b) point toward the presence of domains having a lateral size between 10 and 100 nm. The nature of these domains can be deduced by analyzing the electron diffraction and HRTEM data more carefully. In the case of the growth on a (100)_s_-oriented SrTiO_3_ substrate, two different HRTEM contrast patterns, i.e., domain types of the T-Nb_2_O_5_ film, were observed in images recorded in the [001]s zone axis. Since the HRTEM image shown in Figure 6e was recorded with a small underfocus of Δf ≈ −6 nm and a thickness of the TEM specimen of t ≈ 6 nm, the dark intensity minima in the film correspond to Nb-atomic columns. One type of domain appears in the HRTEM micrograph with a quasi-hexagonal pattern (see right part of Figure 6e). A simplified model of T-Nb_2_O_5_ adapted from Lee et al. [12] (Figure 1c) in the [001]f projection matches the HRTEM pattern very well. This type of domain thus grows with the epitaxial relationship [001]f parallel to [001]s. The second type of domain is characterized by a rectangular HRTEM pattern (see left part of Figure 6e), which matches very well to a model of T-Nb_2_O_5_ being rotated in-plane by 90° with respect to the first type of domain, i.e., the epitaxial relationship of these domains is [001]f perpendicular to [001]s. In the case of growth on (110)s-oriented substrates, only one type of domain was observed, namely, the one with the epitaxial relationship [001]f parallel to [001]s (see Figure 6f). Analyzing the Fourier transformation (not shown here) of that HRTEM image, we found that while the pure in-plane Fourier components of the neighboring domains (left and right side in Figure 6f) match with each other, their out-of-plane Fourier components are separated. This can be explained by a 180° in-plane rotation between the domains. The observed separation of the out-of-plane Fourier components corresponds to a tilt of the (180)_f_ planes of the neighboring domains by approximately 2° with respect to each other. This, in fact, agrees very well with the results of the XRD measurements. The presence of variants of each domain type that are rotated in-plane by 180° leads finally to four and two possible differently oriented domains for T-Nb_2_O_5_ films grown on (100)_s_ and (110)_s_ SrTiO_3_ substrates, respectively.

These findings from the HRTEM analysis are also confirmed on a macroscopic scale by electron diffraction data recorded close to the [001]s zone axis. In the case of a (110)_s_ SrTiO_3_ substrate, the diffraction image of the T-Nb_2_O_5_ film (see Figure 6c) shows only a quasi-hexagonal pattern. For the film grown on a (100)_s_-oriented substrate, there are additional reflections arranged in a rectangular way (mark by red arrows in Figure 6d) in the diffraction image. The weaker spots that appear in both diffraction images and that are split correspond to a periodic superstructure of approx. 3 nm in the films, which is consistent with previous observations [7].

## 4. Summary and Conclusions

We have shown that stable growth conditions can be realized for the epitaxial growth of T-Nb_2_O_5_ on different SrTiO_3_ surfaces by means of MOCVD. Moreover, the number of deposited in-plane domains could be reduced from four to two by the application of (110)_s_-oriented SrTiO_3_. This shows that heteroepitaxial growth can be used to improve the structural properties of T-Nb_2_O_5_ thin films. However, the lattice constant mismatch, as well as the more serious symmetry mismatch between the perovskite substrate and T-Nb_2_O_5_ film, make it challenging to achieve well-ordered epitaxial growth. So far, the growth of single crystalline films on (110)_s_ SrTiO_3_ is hindered by the possible double orientation of nonequivalent 180° in-plane rotated Nb_2_O_5_ domains. Using substrates with an appropriate miscut might be a way to prevent the formation of the two in-plane domain variants and, therefore, to achieve heteroepitaxial growth of single crystalline T-Nb_2_O_5_ films.
